# Curdione protects vascular endothelial cells and atherosclerosis via the regulation of DNMT1-mediated ERBB4 promoter methylation

**DOI:** 10.1515/med-2025-1223

**Published:** 2025-07-14

**Authors:** Yingbiao Wu, Can Jin, Luoning Zhu, Xiaogang Zhang, Xinpeng Cong, Budian Xing, Zhongping Ning

**Affiliations:** Department of Cardiology, Zhoupu Hospital Affiliated to Shanghai Medical College of Health, Shanghai, 201318, China; Department of Cardiology, Zhoupu Hospital Affiliated to Shanghai Medical College of Health, 1500 Zhouyuan Road, Pudong New Area, Shanghai, 201318, China

**Keywords:** atherosclerosis, curdione, DNMT1, ERBB4, HUVECs, ox-LDL

## Abstract

Atherosclerosis (AS) is initiated by the activation of the endothelial cells, which is followed by a series of events that trigger the narrowing of blood vessels and the activation of inflammation. This study aimed to investigate *in vitro* the roles and underlying mechanisms of curdione in AS. Human umbilical vein endothelial cells (HUVECs) were stimulated with oxidized low-density lipoprotein (ox-LDL) and then treated with curdione, after which the growth of the HUVECs and the related mechanisms were determined. HUVECs with ERBB4 overexpression were constructed to explore the role of ERBB4 in curdione-mediated AS. The interaction among ERBB4, methylation, and curdione was confirmed by chromatin immunoprecipitation (ChIP)-quantitative PCR (qPCR) and dual luciferase reporter gene assays. Both curdione and ERBB4 overexpression individually and significantly enhanced viability and proliferation while suppressing apoptosis of the ox-LDL-induced HUVECs, and the combination of curdione and ERBB4 overexpression had better effects. Compared with the ox-LDL-induced HUVECs, both curdione and ERBB4 overexpression individually decreased the levels of IL-6, IL-1β, and IL-8 (*P* < 0.05). They also upregulated Bax, caspase-3, E-cadherin, and F-actin while downregulating Bcl-2 and VEGF (*P* < 0.05). Additionally, the ERBB4 bound to the DNMT1 gene, and the curdione participated in AS via the ERBB4 gene. The study demonstrated that either curdione or ERBB4 overexpression individually may ameliorate AS development by inhibiting apoptosis, inflammation, and the EndMT of HUVECs. In addition, curdione may protect the vascular endothelial cells and AS by regulating the DNMT1-mediated ERBB4 promoter methylation.

## 1 Introduction

Cardiovascular disease (CVD) ranks as the leading global cause of morbidity and mortality and was estimated to have caused 18.6 million deaths in 2019 [1]. It has been reported that there is a significant gender difference in the incidence rate of CVD [2]. For example, the incidence rate of CVD in women is lower in men; however, the CVD incidence and related mortality of postmenopausal women are gradually increasing, such that the incidence in women aged 65–70 years is comparable to that of men [3,4].

Atherosclerosis (AS) is the process of lipid plaque formation in the vascular system, and it is a major risk factor for CVD [5]. AS is initiated by the activation of the endothelial cells (through injury and apoptosis) followed by a series of events (e.g., the accumulation of lipid and fiber elements and calcification) that trigger the narrowing of the blood vessels and the activation of inflammation [6]. The resulting atherosclerotic plaques and these processes can contribute to cardiovascular complications. Hence, the progression of AS is largely dependent on factors such as dyslipidemia, hypertension, and inflammatory response [7]. However, therapeutic drugs (such as statins) developed to target these progressive factors have not achieved satisfactory results; they also carry a significant residual risk of CVD and a wide variety of side effects [8]. Therefore, it is critical to search for new therapeutic strategies to prevent and treat AS.

Herbal medicines have broad-spectrum antibacterial, anti-inflammatory, and anticancer activities, and they are widely used for the prevention and treatment of various diseases due to their significant therapeutic effects and strong safety profile [9]. In recent years, with the progress and development of society, these natural herbal medicines have become popular worldwide, and herbal medicines as supplementary and alternative therapies have been widely accepted in many countries.

At the same time, increasing amounts of evidence have shown that herbal medicines have great potential in the treatment of AS. For example, Zheng et al. [10] showed that Yin-xing-tong-mai decoction could enhance cholesterol efflux by activating the PPARγ-LXRα-ABCA1/ABCG1 pathway, thus alleviating AS. Another study found that the Chinese herbal compound Xinmaikang could mediate macrophage mitochondrial autophagy through the PINK1/Parkin signaling pathway and could be effective against AS [11].

In addition, herbal monomer components, such as natural flavonoids extracted from the herbs *Coptis chinensis* and *Panax notoginseng*, are utilized for AS treatment due to their lipid-lowering and anti-inflammatory effects [12]. Curdione is a sesquiterpenoid component isolated from the essential oil of *Curcuma aromatica* Salisb., which has beneficial properties for combating inflammation, oxidative stress, tumor growth, and fungal infections [13]. A previous study demonstrated that curdione could inhibit the viability and proliferation of uterine leiomyosarcoma cells in a concentration- and time-dependent manner, and its anti-tumor effects could be targeted by IOD1 [14]. An investigation by Wang et al. [15] revealed that curdione induced m6A methylation-mediated ferroptosis through the YTHDF2 gene and the METTL14 gene in colorectal cancer cells and thus may be a promising approach for treating colorectal cancer. These reports imply that curdione may play a protective role against various diseases. However, the roles and underlying mechanisms of curdione in CVD (such as AS) remain unknown.

DNA methylation induces changes in gene expression without altering the DNA sequence by adding methyl groups to cytosine in nucleotides containing CpG, thereby forming 5-methylcytosine 5 [16]. AS is an epigenetic disease, and emerging research has shown that abnormal DNA methylation plays a pivotal role in the inflammatory response, endothelial damage, foam cell formation, and smooth muscle cell proliferation [17]. Therefore, we believe that DNA methylation is closely related to the occurrence and development of AS. In addition, previous studies demonstrated that single compounds and herbal preparations can modulate DNA methylation in AS [18,19].

ERBB4 is a member of the ERBB family of receptor tyrosine kinases, and it contains a CpG island. ERBB4 can bind to neuromodulatory protein (NRG), and it can be activated by other factors as well as induce a range of cellular responses, such as cell division and cell differentiation [20]. Huang et al. showed that icariin could relieve AS and inhibit the proliferation and migration of oxidized low-density lipoprotein (ox-LDL)-induced human aortic vascular smooth muscle cells via the signaling of miR-205-5p/ERBB4/AKT [21]. Another study found that miR-205-5p could suppress the viability and migration of ox-LDL-induced human umbilical vein endothelial cells (HUVECs) by regulating ERBB4/AKT signaling [22]. These findings suggested that ERBB4 may play an important role in AS occurrence and progression. However, whether curdione can affect the development of CVD (such as AS) via modulating ERBB4 promoter methylation is unclear.

ox-LDL is the primary inducer of AS. In addition to triggering oxidative stress and damaging cells, ox-LDL is thought to promote endothelial dysfunction and accelerate the proliferation of cells involved in AS etiology, such as monocytes, smooth muscle cells, and endothelial cells [23]. Multiple studies have reported that ox-LDL can facilitate the apoptosis of HUVECs and regulate the activity of caspase-3 and caspase-9, thus playing a key role in the pathogenesis of CVD [24,25]. Therefore, in this study, HUVECs were stimulated with ox-LDL to establish an AS model *in vitro* and then treated with curdione to investigate the effects of curdione on AS progression. Next, HUVECs with ERBB4 overexpression were constructed, and the roles and potential mechanisms of ERBB4 in the regulating of AS by curdione were further explored. Our work will provide novel and valuable candidates for the treatment of AS and other forms of CVD.

## 2 Materials and methods

### 2.1 Cell culture and grouping

HUVECs were obtained from Shanghai Zhong Qiao Xin Zhou Biotechnology Co., Ltd. (Shanghai, China) and were maintained in Dulbecco’s Modified Eagle’s Medium (Thermo Fisher Scientific, Waltham, MA USA) containing 10% fetal bovine serum (Thermo Fisher Scientific) and 1% penicillin/streptomycin (Thermo Fisher Scientific). The cells were cultured in an incubator with 5% CO_2_ at 37°C.

To explore the role of curdione in AS, HUVECs were divided into four groups as follows (*n* = 3): a control group, the ox-LDL group, the ox-LDL + PBS group, and the ox-LDL + CUR group. The cells in the control group were not given any treatment, and the cells in the other three groups were first induced by 100 mg/mL ox-LDL (Solarbio, Beijing, China) for 12 h. Then, the cells in the ox-LDL group were not given any treatment, while the cells in the ox-LDL + PBS and ox-LDL + CUR groups were administered equal amounts of phosphate buffered saline (PBS) solution and 100 μM curdione (CUR) [14], respectively, for 24 h.

To further investigate the potential mechanism of ERBB4 in the curdione regulation of AS progression, the HUVECs were randomly assigned into five groups (*n* = 3): control, ox-LDL, ox-LDL + NC, ox-LDL + OE-ERBB4, and ox-LDL + + OE-ERBB4 + CUR.

### 2.2 Cell transfection

The negative control (NC, pEGFP-N2 empty plasmids) and OE-ERBB4 (pEGFP-N2-ERBB4 plasmids) were designed, synthesized, and provided by GENEray Biotechnology (Shanghai, China). The methods of cell transfection were shown as previously described [26]. HUVECs were seeded into a 24-well plate at a density of 5 × 10^5^ cells/well, and, after being cultured to an approximately 80% confluence, the culture medium was changed to a free-serum medium. Then, the HUVECs were transfected with 4 μm pEGFP-N2 empty plasmids or pEGFP-N2-ERBB4 plasmids using Lipofectamine 2000 (Thermo Fisher Scientific). After 6 h of transfection, the medium was replaced with the complete medium. After culturing for another 24 h, the cells were harvested to isolate total RNA. Western blot was employed to measure the ERBB4 expression to evaluate the efficiency of the cell transfection.

### 2.3 Cell proliferation assay

The viability and proliferation of the HUVECs with different treatments were measured using the Cell Counting Kit-8 (CCK8; Beyotime Biotechnology, Shanghai, China) and BeyoClick™ EdU Cell Proliferation Kit with Alexa Fluor 488 (Beyotime Biotechnology) in accordance with the manufacturer’s instructions. For cell viability, the cells with different treatments were harvested and added with 10 μL CCK8 reagent. After culturing for about 2 h, a microplate reader (Thermo Fisher Scientific) was applied to determine the absorbance at 450 nm, and cell viability was calculated.

For cell proliferation, the HUVECs with different treatments were obtained and added with 10 μM EdU solution. After incubating at 37°C for 2 h, the cells were fixed with 4% paraformaldehyde for 15 min and then permeabilized with 0.3% Triton X-100 for 15 min. After washing, the cells were added with 0.5 mL Click reaction solution and incubated at room temperature for 30 min in the dark. After washing, 1 mL of 1× Hoechst 33342 (5 μg/mL) was added to stain the cell nucleus for 10 min in the dark. After washing again, the cells were acquired under a fluorescence microscope (Thermo Fisher Scientific).

### 2.4 Cell apoptosis assay

The apoptosis of the HUVECs was determined using an Annexin V-FITC Apoptosis Assay Kit (Beyotime Biotechnology) and a One-Step TUNEL Apoptosis Assay Kit (Beyotime Biotechnology) following the supplier’s instructions. For the Annexin V-FITC Apoptosis Assay Kit, the harvested HUVECs with different treatments were centrifuged at 1,000 rpm for 5 min and resuspended with Annexin V-FITC binding solution. Then, the cells were added with 10 μL Annexin V-FITC and 5 μL propidium iodide. After 15 min of incubation in the dark, the pictures of the HUVECs were observed under flow cytometry (Becton, Dickinson and Company, NJ, USA). In addition, the total cell apoptosis rate was analyzed using the CellQuest software (Becton, Dickinson and Company).

For the TUNEL assay, the HUVECs with different treatments were fixed with 4% paraformaldehyde for 30 min and then added with PBS containing 0.3% Triton-X-100 for 5 min. After washing with PBS twice, the cells were added with 50 μL TUNEL detection solution and incubated at 37°C in the dark for 60 min. After washing with PBS three times, 1 mL of 1× Hoechst 33342 solution was added to the cells for 10 min in the dark. After washing, a fluorescence microscope was employed to observe the cells after sealing them with the anti-fluorescence quenching sealing solution.

### 2.5 Enzyme-linked immunosorbent assay

The HUVECs with different treatments were acquired and centrifuged at 200 × *g* for 5 min. The cell supernatant was collected to determine the concentrations of IL-1β, IL-6, and IL-8 in the HUVECs using the enzyme-linked immunosorbent assay (ELISA) with the Human IL-1β ELISA Kit (Beyotime Biotechnology), the Human IL-6 ELISA Kit (Beyotime Biotechnology), and the Human IL-8 ELISA Kit (Beyotime Biotechnology), respectively, in line with the recommendations of the manufacturer.

### 2.6 Real-time qPCR (RT-qPCR)

Total RNA was extracted from the different HUVECs using TRIzol reagent with the manufacturer’s protocol, after which it was quantified and qualified using a microplate reader. The qualified RNA was reverse transcribed into Cdna using the PrimeScriptRT Reagent Kit (Takara). Then, RT-qPCR was initiated at 50°C for 2 min and at 95°C for 2 min, followed by 40 cycles at 95°C for 3 s and 60°C for 30 s. The melting procedure was conducted at 95°C for 15 s, 60°C for 60 s, and 95°C for 15 s. The mRNA expression of the related genes was examined using the 2^−ΔΔCt^ method with GAPDH as the internal reference. The sequences of ERBB4 were shown as F, 5′-GAACAGCAGTACCGAGCCTT-3′ and R, 5′- GCAACGTCCACATCCTGAAC-3′. The sequences of GAPDH were shown as F, 5′- GCAACTAGGATGGTGTGGCT-3′ and R, 5′- TCCCATTCCCCAGCTCTCATA-3′.

### 2.7 Western blot

The HUVECs were obtained to isolate the total protein with the RIPA Lysis and Extraction Buffer (Thermo Fisher Scientific), and the concentrations of the isolated proteins were measured using a BCA protein assay kit. Thereafter, the protein samples (20 μg) were separated with 10% SDS-PAGE (70 V for 30 min and 120 V for 60 min) and transferred to PVDF membranes (250 mA for 90 min). Next, they were blocked with 5% skim milk for e hour, and the membranes were incubated with the primary antibodies at 4°C overnight, after which they were incubated with the HRP-conjugated secondary antibodies (1: 1,000; HUABIO).

After they were cultured at 37°C for 2 h, the protein bands were visualized by the Enhanced Chemiluminescence Assay Kit (Beyotime Biotechnology). The primary antibodies included the anti-Bax antibody (1: 1,000, HUABIO), the anti-Bcl-2 antibody (1: 1,000, HUABIO), the anti-caspase-3 antibody (1: 1,000, HUABIO), the anti-GAPDH antibody (1: 1,000, HUABIO), the anti-E-cadherin antibody (1: 1,000, HUABIO), the anti-F-actin antibody (1: 1,000, Abcam), the anti-VEGF antibody (1: 1,000, HUABIO), the anti-ERBB4 antibody (1: 1,000, HUABIO), the anti-DNMT1 antibody (1: 1,000, HUABIO), the anti-DNMT3a antibody (1: 1,000, HUABIO), and the anti-DNMT3b antibody (1: 1,000, HUABIO).

### 2.8 ChIP-qPCR

In accordance with the manufacturer’s protocols, a ChIP assay kit (Beyotime Biotechnology) was used for the ChIP-qPCR. Briefly, the HUVECs (1 × 10^6^ cells) were fixed with 1% formaldehyde at 37°C for 10 min, after which the fixed cells were collected, lysed, and sonicated for 15 cycles of 10 s on/10 s off with 50% power using an Ultrasonic Cell Crusher (JY92-IIN, Beijing Huilong Environmental Instruments Co., Ltd., Beijing, China). The anti-DNMT1 antibody (HUABIO) and rabbit IgG (HUABIO) were employed for immunoprecipitation. The precipitated DNA samples were purified and amplified by qPCR. The sequences of ERBB4 for the ChIP-qPCR assay were left: AATTTTTTTGTGGGTTGTAGTTG and right: ATTCTACTCCTTCTTCAATTTCCTTAC.

### 2.9 Dual-luciferase reporter gene assay

The plasmids of pGL3-basic and pGL3-ERBB4-WT were synthesized, constructed, and provided by Yanzai Biotechnology Co. Ltd. (Shanghai, China). Briefly, the HUVECs were seeded into a 24-well plate at a density of 1 × 10^5^ cells/well and cultured overnight. Afterwards, pGL3-basic (0.4 μg), pGL3-ERBB4-WT (0.4 μg), and pGL3-ERBB4-MUT (0.4 μg) were co-transfected to the cells with si-NC (100 nM) or si-DNMT1 (100 nM) using Lipofectamine 2000 (Thermo Fisher Scientific) following the supplier’s protocols. After culturing for another 24 h, the cells were treated with PBS or curdione (100 μM) for 24 h. Finally, the relative luciferase activity in the different groups was determined with a dual luciferase reporter system (Promega, WI, USA).

### 2.10 Statistical analysis

The data were expressed as mean ± standard deviation. The SPSS software was used to perform all of the statistical analyses, and GraphPad Prism 5 was used for picture drawing. For the comparison between the two groups, Student’s *t*-test was applied. One-way analysis of variance followed by Tukey’s HSD was employed to compare more than two groups. A *P*-value of less than 0.05 was determined to represent a statistically significant difference.

## 3 Results

### 3.1 The effects of curdione on the growth of ox-LDL-induced HUVECs

**Figure 1:**
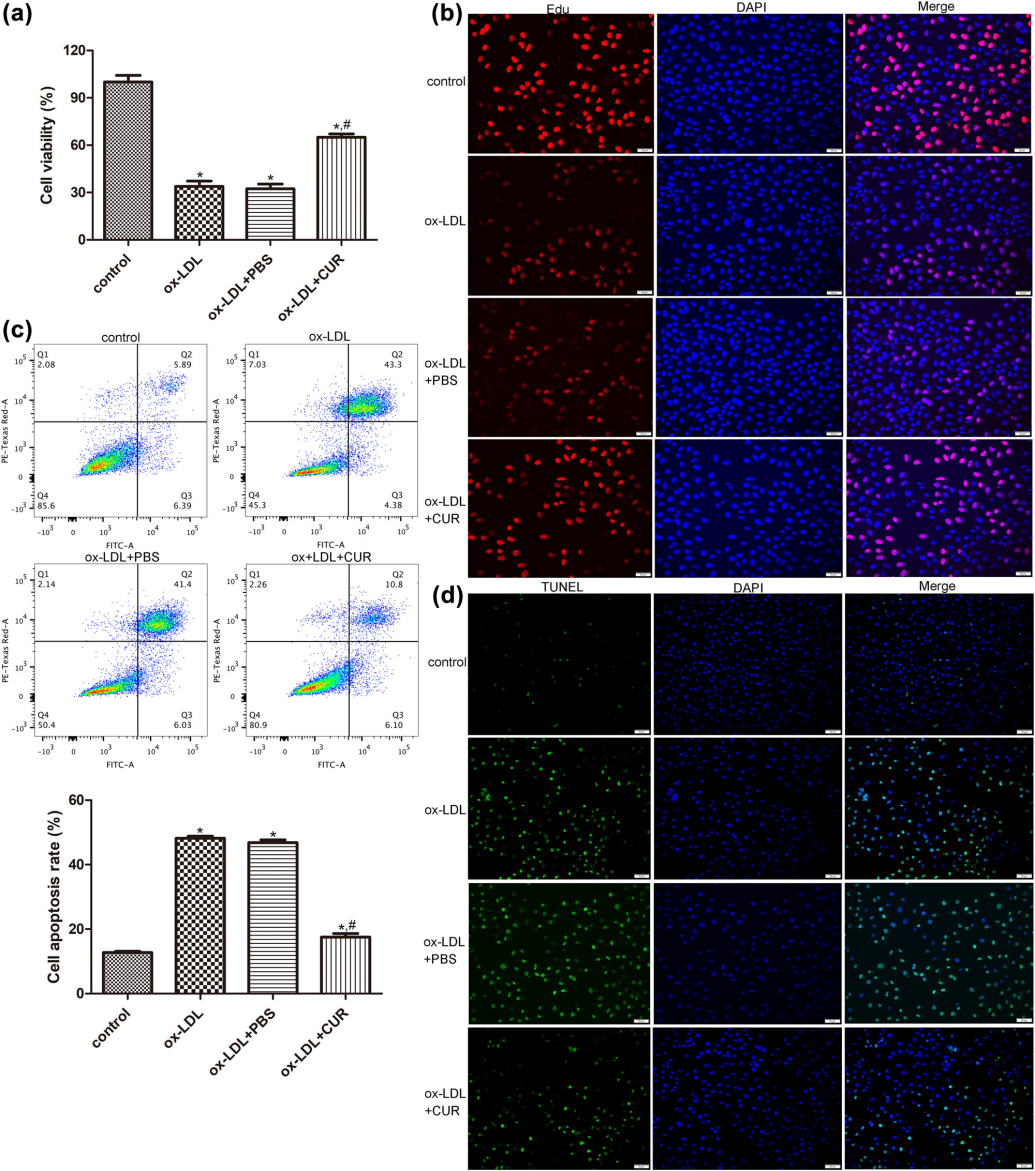
Effects of curdione on the growth of oxidized low-density lipoprotein (ox-LDL)-induced HUVECs. (a) The viability of HUVECs treated with ox-LDL and curdione determined by cell counting kit-8. (b) The proliferation of HUVECs treated with ox-LDL and curdione determined by EdU assay. The apoptosis of HUVECs treated with ox-LDL and curdione measured by flow cytometry (c) and TUNEL assay (d). CUR: Curdione. *P < 0.05 vs control; ^#^ *P* < 0.05 vs ox-LDL.

**Figure 2:**
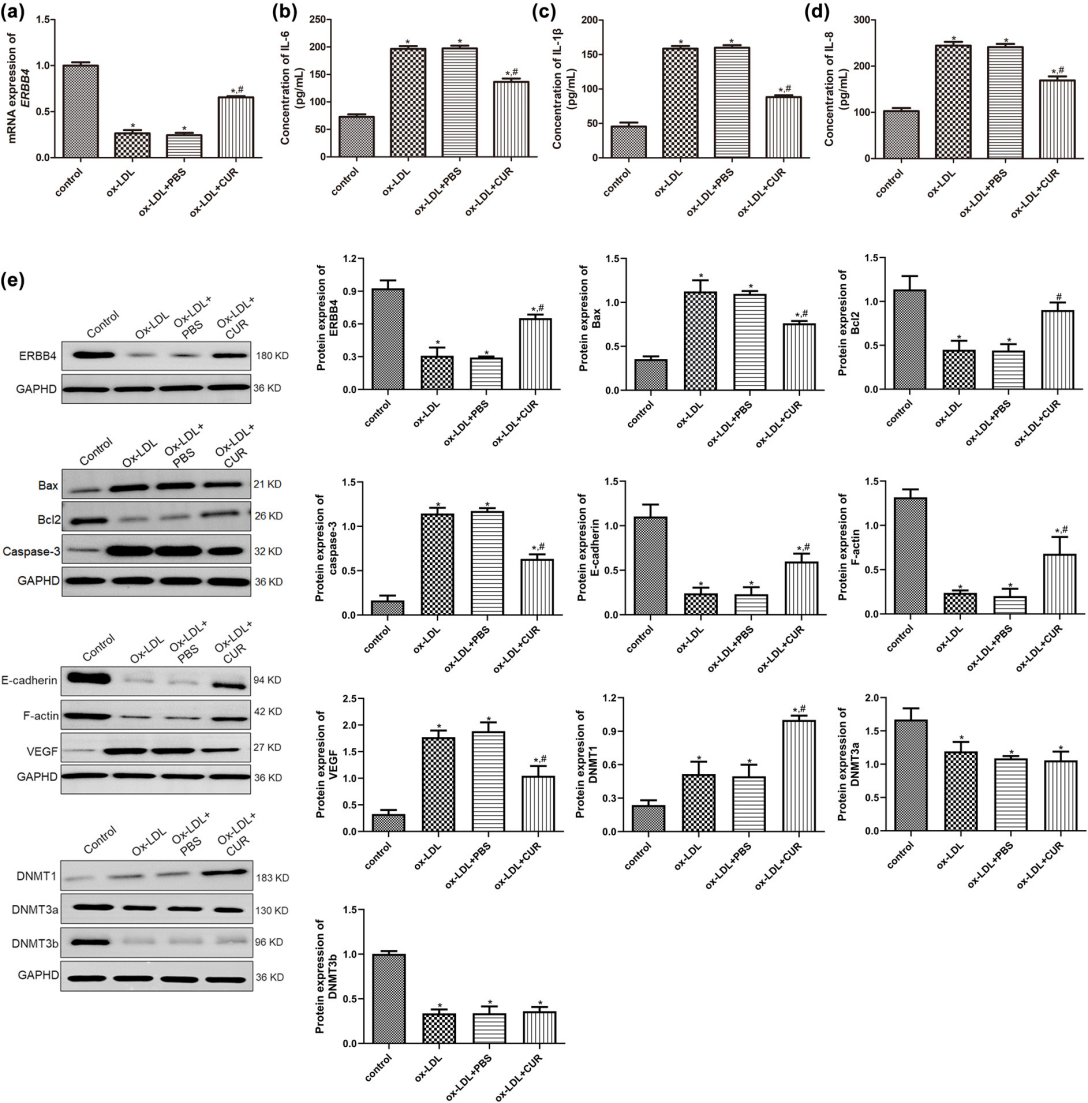
Effects of curdione on the concentrations of inflammatory cytokines, and expression of apoptosis-related, endothelial-mesenchymal transition (EndMT)-related, and DNA methylation-related proteins. (a) The mRNA expression ERBB4 in the HUVECs treated with ox-LDL and curdione using real-time quantitative PCR. The concentrations of IL-6 (b), IL-1β (c), and IL-8 (d) in the HUVECs treated with ox-LDL and curdione by ELISA. (e) The protein expression of ERBB4, apoptosis-related (Bax, Bcl-2, and caspase-3), EndMT-related (E-cadherin, F-actin, and VEGF), and DNA methylation-related proteins (DNMT1, DNMT3a, and DNMT3b) in the HUVECs treated with ox-LDL and curdione using Western blot. CUR: Curdione. *P < 0.05 vs control; ^#^ *P* < 0.05 vs ox-LDL.

To investigate the role of curdione in AS progression, the HUVECs were stimulated with ox-LDL and then treated with curdione. Cell viability and apoptosis were then determined. There were no significant differences in terms of the viability and apoptosis of the HUVECs between the ox-LDL and ox-LDL + PBS groups (*P* > 0.05, e). The CCK8 results showed that ox-LDL induction significantly inhibited the viability of the HUVECs compared with the control cells (*P* < 0.05), while the curdione treatment was seen to enhance the viability of the ox-LDL-induced HUVECs (*P* < 0.05, Figure 1a). The tendency of cell proliferation in the different groups determined by EdU assay was similar with that of cell viability measured by CCK8 (Figure 2b). Additionally, the outcomes of the flow cytometry and the TUNEL assay revealed that, compared with the control cells, the apoptosis rate was remarkedly increased in the ox-LDL-induced HUVECs (*P* < 0.05), whereas it was markedly decreased after the curdione treatment (*P* < 0.05, Figure 1c and d). These results indicated that curdione promoted the viability and proliferation of the ox-LDL-induced HUVECs while suppressing their apoptosis.

### 3.2 Effects of curdione on the expression of apoptosis-related, EndMT-related, and DNA methylation-related proteins

We further determined the mRNA and protein expression of ERBB4 in the HUVECs with different treatments and found that ERBB4 mRNA and protein expression were significantly downregulated after ox-LDL stimulation compared to the control HUVECs (*P* < 0.05), while it was upregulated in the ox-LDL + CUR group relative to the ox-LDL group (*P* < 0.05, Figure 2a and e), which suggested that ERBB4 was significantly upregulated after curdione administration.

Further, to explore the underlying mechanisms of the curdione regulation of the ox-LDL-induced HUVECs, the concentrations of inflammatory cytokines (IL-6, IL-1β, and IL-8), as well as the expressions of apoptosis-related proteins (Bax, Bcl-2, and caspase-3), endothelial–mesenchymal transition (EndMT)-related proteins (E-cadherin, F-actin, and VEGF), and DNA methylation-related proteins (DNMT1, DNMT3a, and DNMT3b) in the different groups, were detected. The concentrations of IL-6 in the control, ox-LDL, ox-LDL + PBS, and ox-LDL + CUR groups were, respectively, 72.84 ± 4.58, 196.54 ± 4.97, 197.36 ± 5.12, and 136.49 ± 6.15 pg/mL, which indicated that ox-LDL induction significantly enhanced the IL-6 concentration compared with the control cells (*P* < 0.05). At the same time, the curdione reduced its level caused by ox-LDL (*P* < 0.05, Figure 2b). Moreover, the trend of the IL-1β and IL-8 concentrations in the HUVECs with different administrations was consistent with that of the IL-6 level (Figure 2c and d). These results implied that curdione inhibited the inflammation in the HUVECs caused by ox-LDL.

The Western blot results showed that in comparison with the control HUVECs, the protein expression of Bax, caspase-3, and VEGF was significantly higher in the ox-LDL-induced HUVECs (*P* < 0.05), while the curdione treatment appeared to reverse their expression induced by ox-LDL (*P* < 0.05, Figure 2e). The protein expression of Bcl-2, E-cadherin, and F-actin was significantly lower after ox-LDL stimulation relative to the control cells (*P* < 0.05), whereas it was higher after the addition of curdione compared with the ox-LDL-induced HUVECs (*P* < 0.05, Figure 2e). Furthermore, compared with the control group, the expression of DNMT1 was significantly upregulated after ox-LDL induction (*P* < 0.05), and the curdione further markedly its expression (*P* < 0.05, Figure 2e). However, for DNMT3a and DNMT3b, their protein expression was significantly downregulated in the ox-LDL-treated HUVECs compared to the control cells (*P* < 0.05). Moreover, the curdione did not appear to change their expression caused by ox-LDL (*P* > 0.05, Figure 2e).

### 3.3 Cell transfection efficiency

**Figure 3:**
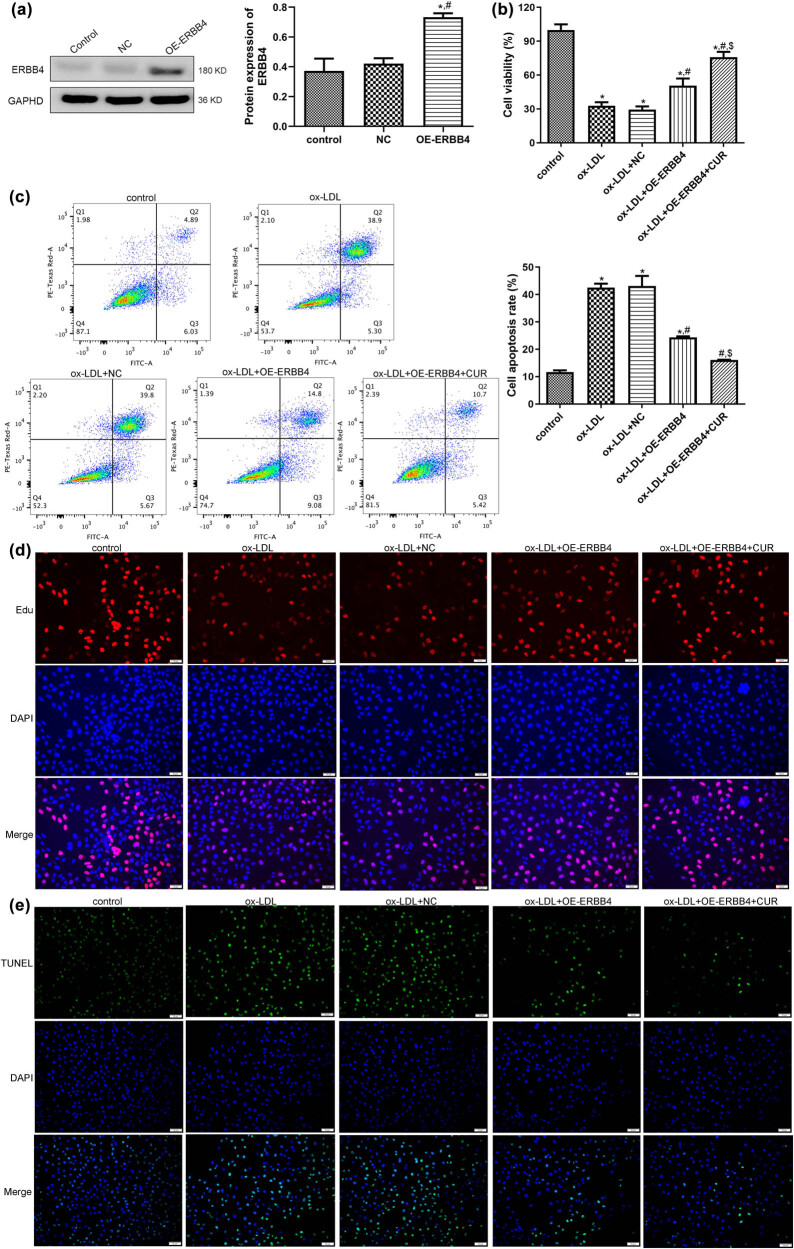
Effects of ERBB4 on the growth of curdione regulating ox-LDL-induced HUVECs. (a) Evaluation of cell transfection efficiency through determining the expression of ERBB4 using Western blot. (b) The viability of HUVECs after OE-ERBB4 transfection via cell counting kit-8. (c) The apoptosis of HUVECs after OE-ERBB4 transfection measured by flow cytometry. (d) The proliferation of HUVECs after OE-ERBB4 transfection examined by EdU assay. (e) The apoptosis of HUVECs after OE-ERBB4 transfection measured by TUNEL assay. CUR: Curdione. *P < 0.05 vs control; ^#^ *P* < 0.05 vs ox-LDL; ^$^ *P* < 0.05 vs ox-LDL + OE-ERBB4.

Due to the higher expression of ERBB4 after being treated with curdione in comparison with the ox-LDL group, to further reveal the role of ERBB4 in AS development, HUVECs with ERBB4 overexpression were first established, and the cell transfection efficiency was assessed. No significant difference was observed in the ERBB4 protein expression between the control and NC groups (*P* > 0.05). However, the ERBB4 protein expression was significantly enhanced after transfection with OE-ERBB4 (*P* < 0.05, Figure 3a), which implied that the HUVECs with ERBB4 overexpression were successfully constructed and could be used for subsequent experiments.

### 3.4 Effects of ERBB4 on the growth of curdione-regulated, ox-LDL-induced HUVECs

The constructed HUVECs with ERBB4 overexpression were first induced by ox-LDL and then treated with curdione to further study the effects of ERBB4 on the growth of ox-LDL-induced HUVECs. It was observed that the viability of the HUVECs was significantly inhibited by ox-LDL compared with the control HUVECs (*P* < 0.05). The ERBB4 overexpression apparently enhanced the viability of the ox-LDL-induced HUVECs (*P* < 0.05), and it further promoted the viability of the ox-LDL-induced HUVECs with ERBB4 overexpression (*P* < 0.05, Figure 3b). In comparison with the control HUVECs, ox-LDL significantly increased the apoptosis of the HUVECs (*P* < 0.05), whereas their apoptosis was apparently inhibited by ERBB4 overexpression (*P* < 0.05). At the same time, the combination of curdione and ERBB4 overexpression further markedly reduced the apoptosis of the ox-LDL-induced HUVECs (*P* < 0.05, Figure 3c). Additionally, the tendency of cell proliferation and apoptosis in the different groups examined by the EdU (Figure 3d) and TUNEL assays (Figure 3e) were in line with the cell viability and apoptosis tested by CCK8 and the flow cytometry. These outcomes revealed that ERBB4 overexpression combined with curdione further promoted the viability and proliferation of the ox-LDL-induced HUVECs while further repressing their apoptosis.

### 3.5 Effects of ERBB4 on the expression of inflammatory cytokines and apoptosis-related and EndMT-related proteins in curdione-regulated, ox-LDL-induced HUVECs

**Figure 4:**
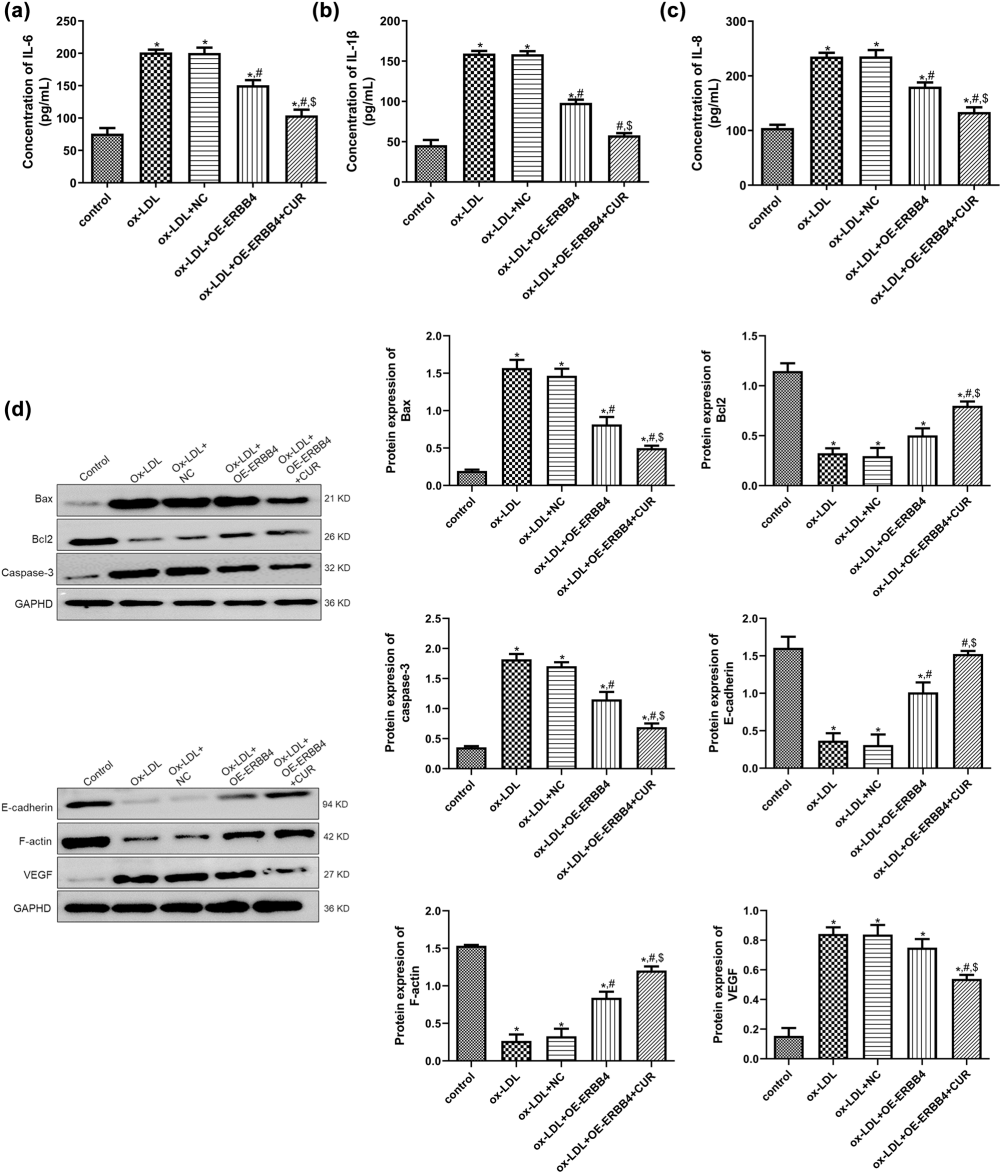
Potential mechanisms of ERBB4 in curdione regulating ox-LDL-induced HUVECs. The concentrations of IL-6 (a), IL-1β (b), and IL-8 (c) in HUVECs after OE-ERBB4 transfection determined by ELISA. (d) The protein expression of ERBB4, apoptosis-related (Bax, Bcl-2, and caspase-3), and EndMT-related (E-cadherin, F-actin, and VEGF) in HUVECs after OE-ERBB4 transfection detected by Western blot. CUR: Curdione. *P < 0.05 vs control; ^#^ *P* < 0.05 vs ox-LDL; ^$^ *P* < 0.05 vs ox-LDL + OE-ERBB4.

Next, the potential mechanism of ERBB4 in the curdione regulation of the ox-LDL-induced HUVECs was investigated. For the inflammatory cytokines IL-6, IL-1β, and IL-8, it was shown that their levels were significantly higher in the ox-LDL-induced cells than in the control cells (*P* < 0.05), but ERBB4 overexpression reduced their levels caused by ox-LDL (*P* < 0.05), and ERBB4 overexpression combined with ERBB4 further decreased their levels (*P* < 0.05, Figure 4a–c). For the apoptosis-related proteins Bax, Bcl-2, and caspase-3, compared with the control cells, ox-LDL stimulation significantly upregulated the protein expression of Bax and caspase-3, while it downregulated Bcl-2 (*P* < 0.05). In addition, ERBB4 overexpression evidently reversed the protein expression caused by the ox-LDL (*P* < 0.05), and ERBB4 overexpression further reversed the expression induced by ox-LDL (*P* < 0.05, Figure 4d). In addition, the trend of E-cadherin and F-actin protein expression in the different groups was opposite that of the Bax protein expression (Figure 4e). For the VEGF, ox-LDL significantly upregulated its expression compared with the control group (*P* < 0.05). At the same time, ERBB4 combined with curdione markedly downregulated the expression caused by the ox-LDL (*P* < 0.05, Figure 4e).

### 3.6 Interaction between ERBB4 and DNMT1

**Figure 5:**
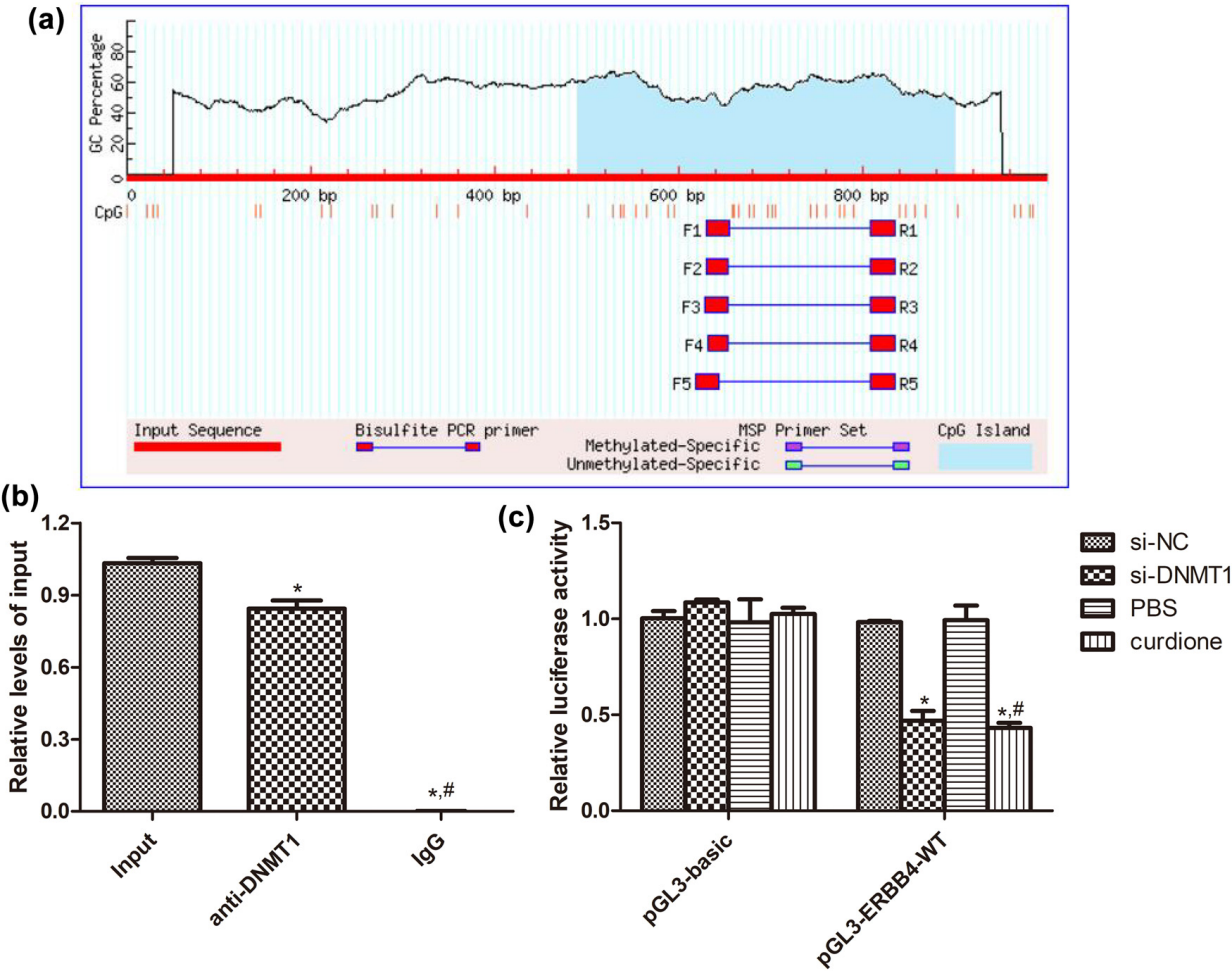
Interaction between ERBB4 and DNMT1. (a) ERBB4 had the CpG island based on the MethPrimer database. (b) ChIP-qPCR was employed to detect the binding of DNMT1 to the ERBB4 promoter in HUVECs. IgG was applied as an NC. *P < 0.05 vs input; ^#^ *P* < 0.05 vs anti-DNMT1. (c) The interaction between ERBB4 and DNMT1; as well as between ERBB4 and curdione determined using dual luciferase reporter gene assay. *P < 0.05 vs si-NC; ^#^ *P* < 0.05 vs PBS.

Based on the MethPrimer database, we observed that ERBB4 had a CpG island, which may be the binding site of DNMT1 (Figure 5a). After that, we used ChIP-qPCR and the dual luciferase reporter gene assay to validate the interaction between ERBB4 and DNMT1. The results of the ChIP-qPCR confirmed that ERBB4 could bind to the promoter region of DNMT1 (Figure 5b). In addition, the dual luciferase reporter gene assay revealed that in the pGL3-basic plasmid, there were no significant differences in relative luciferase activity among the si-NC, si-DNMT1, PBS, and curdione groups (*P* > 0.05, Figure 5c). However, in the pGL3-ERBB4-WT plasmid, compared with the si-NC group of the PBS group, the relative luciferase activity in the si-DNMT1 and curdione groups was significantly decreased (*P* < 0.05, Figure 5c). These indicated that ERBB4 could bind to DNMT1, and curdione could play an important role in AS via the regulation of ERBB4.

## 4 Discussion

AS is a chronic disease of inflammation and lipid deposition that ultimately leads to acute cardiovascular events, which seriously threaten health and life [27]. Curdione is the active ingredient of the traditional Chinese medicine Curcuma zedoary, and it has a variety of pharmacological effects, such as anti-inflammatory and antioxidant effects and the inhibition of platelet aggregation [28]. The ERBB4 gene has been found to be a promising diagnostic and therapeutic target for metabolic disorders [29]. Our study is the first to explore the roles and underlying mechanisms of curdione in AS development *in vitro* and to further investigate the specific role of ERBB4. We found that curdione could enhance the proliferation and inhibit the apoptosis of ox-LDL-stimulated HUVECs as well as upregulate ERBB4 expression and regulate DNMT1. Afterwards, HUVECs with ERBB4 overexpression were successfully established. Similar to curdione, ERBB4 overexpression significantly improved the growth of the HUVECs induced by ox-LDL. A combination of curdione and ERBB4 overexpression had even better effects. Additionally, we observed that ERBB4 could bind to DNMT1, and curdione could be involved in AS through the regulation of the ERBB4 gene. These results indicated that curdione may protect AS through DNMT1-mediated ERBB4 promoter methylation.

It has been confirmed that endothelial cell injury and apoptosis play major roles in the pathogenesis of AS, and oxidative injury is an important risk factor for endothelial cell injury [30]. The loss of the morphological and functional integrity of vascular endothelial cells in AS is closely related to cellular inflammation and apoptosis [31,32]. ox-LDL promotes the formation and development of atherosclerotic plaques by inducing endothelial inflammation and apoptosis [33]. Our outcomes showed that ox-LDL induction could inhibit the viability and enhance the apoptosis of HUVECs, increase the concentrations of IL-6, IL-1β, and IL-8, and upregulate Bax and caspase-3 while downregulating Bcl-2. However, curdione and ERBB4 could restore the changes caused by ox-LDL, and a combination of curdione and ERBB4 had better actions. IL-6, IL-1β, and IL-8 are all pro-inflammatory cytokines, and higher levels are strongly associated with increased inflammation in the cells. Apoptosis, a form of programmed cell death, is necessary for normal cell renewal, normal development, and chemically induced cell death. However, abnormal apoptosis contributes to a variety of human diseases, such as cancer and neurodegenerative diseases [34]. Bax, a kind of pro-apoptotic protein, is required to perform additional mitochondrial changes in mitochondrial outer membrane penetration and apoptosis [35]. Upregulation of Bcl-2 has been found to be strongly associated with anti-apoptosis, and Bcl-2 has been reported to participate in vascular endothelial cell damage and AS [36]. Zhu et al. [37] demonstrated that LINC00659 could regulate the apoptosis of HUVECs via mediating the expression of Bax and Bcl-2, thereby playing an important role in deep vein thrombosis. The execution of apoptosis is mediated by the apoptotic caspase family, which accelerates cell death by cutting a defined set of target proteins, thereby resulting in the organized breakdown of cell components [34]. Caspase-3 belongs to the caspase family and is the primary executor of apoptosis. There is growing evidence that caspase-3 also plays a key role in regulating the growth and homeostasis maintenance of normal and malignant cells and tissues in multicellular organisms [38]. A previous study by Lyu et al. [39] illustrated that ginsenoside Rg1 could relieve apoptosis, inflammation, and oxidative stress by regulating the expression of Bax, Bcl-2, and caspase-3 and reducing the levels of IL-1β, IL-6, and TNF-α in ox-LDL-induced HUVECs, thereby laying a foundation for the treatment of CVD with Rg1 in the future. Liu et al. also showed that paeoniflorin could repress the apoptosis and inflammation in human coronary artery endothelial cells stimulated by ox-LDL via decreasing the levels of IL-6 and IL-8, as well as downregulating Bax while upregulating Bcl-2 [40]. Another investigation demonstrated that DUSP12 overexpression could enhance the viability and suppress the apoptosis of ox-LDL-caused HUVECs while upregulating Bcl-2 and downregulating Bax, as well as declining the concentrations of TNF-α, IL-1β, and IL-6, which indicated that DUSP12 may be a target for AS therapy [41]. In consideration of these research findings, we speculate that either curdione or ERBB4 overexpression may promote proliferation while inhibiting apoptosis and inflammation in ox-LDL-induced HUVECs by regulating the inflammatory cytokines IL-6, IL-1β, and IL-8 and the apoptosis-associated proteins Bax, Bcl-2, and caspase-3, thereby ameliorating AS progression. Additionally, a combination of curdione and ERBB4 overexpression could exhibit even better effects compared to the single treatment.

In recent years, EndMT has become increasingly common in AS. It is characterized by changes in the phenotype of normal endothelial cells with the same shape and properties as mesenchymal cells, including enhanced proliferation and migration, secreting extracellular matrix proteins, such as fibronectin and collagen, and expressing various leukocyte adhesion molecules [42]. In the current study, both curdione and ERBB4 overexpression or a combination of curdione and ERBB4 overexpression upregulated E-cadherin and F-actin expression, whereas they downregulated the VEGF expression induced by ox-LDL. E-cadherin, a marker of endothelial cells, is a key component of the adhesive junctions that are integral in cell adhesion and the maintenance of the cell epithelial phenotype [43]. Jiang et al. [44] revealed that miR-449a could induce EndMT by targeting the iteration between AdipoR2 and E-cadherin so as to promote the development of AS. Many fundamental cellular processes, such as endocytosis, division, locomotion, and polarization, need assembly, maintenance, and disassembly of the F-actin networks at specific locations and times within the cell, and the dynamic transformation of F-actin controls cell movement in eukaryotes [45]. A previous exploration showed that naringin could upregulate the expression of VE-cadherin and F-actin caused by ox-LDL in HUVECs as well as restore the endothelial barrier integrity via the inhibition of EndMT of ox-LDL-induced HUVECs, thus protecting the endothelial cells from apoptosis and inflammation and improving AS [46]. VEGF is a key regulator of angiogenesis, lymphangiogenesis, lipid metabolism, and inflammation, and it is involved in the development of AS and further CVD [47]. It has been reported that VEGF can inhibit inflammation, promote the expansion and proliferation of lymphatic vessels, reduce oxidative stress, and thus prevent the progression of AS [48]. Zhang et al. found that protocatechuic aldehyde could promote the proliferation, adhesion, and migration of ox-LDL-induced human microvascular pericytes and downregulate the expression of VEGF-A to reduce ox-LDL-caused pericyte dysfunction, thereby maintaining the structure of the capillary network and the stability of atherosclerotic plaques [49]. These studies, together with our results, imply that both curdione and ERBB4 overexpression may suppress the EndMT of HUVECs caused by ox-LDL via the regulation of E-cadherin, F-actin, and VEGF, thus exerting a protective effect against AS.

In addition, blood flow disorders can lead to AS, whereas stabilizing blood flow prevents AS by differentially regulating gene expression in the endothelial cells [50]. DNA methylation confers persistent changes in gene expression. It also plays a role in the regulation of specific differential genes and has essential functions in terms of maintaining endothelial cell homeostasis and the development of vascular diseases [51]. Our study observed that ERBB4 was upregulated after curdione treatment in the ox-LDL-induced HUVECs, and, after searching the database, we found that ERBB4 contains a CpG island. Therefore, we further discovered the promoter methylation of ERBB4. The establishment and maintenance of DNA methylation in mammals is achieved by two groups of DNA methyltransferases (DNMT), including DNMT3a and DNMT3b, which are responsible for installing DNA methylation patterns during gametogenesis and early embryogenesis, as well as DNMT1, which is critical for spreading DNA methylation patterns during replication [52]. Therefore, we determined the expression of DNMT1, DNMT3a, and DNMT3b in this research and discovered that only DNMT1 was further upregulated after treatment with curdione. The ChIP-qPCR and dual luciferase reporter gene assay results revealed that curdione, ERBB4, and DNMT1 interacted with each other. Similarly, Yang et al. [53] showed that fibroblast ALKBH5 positively regulated healing after myocardial infarction by stabilizing ERBB4 mRNA in an m6A-dependent manner. Meng et al. [54] found that FOXO3a could bind to the promoter region of SPRY2 to promote ZEB1 expression and that MeCP2 could suppress ischemic neuronal damage by heightening the promoter methylation of FOXO3a. Another investigation described how miR-221 could alleviate ox-LDL-induced macrophage inflammation via inhibiting DNMT3B-mediated NCoR promoter methylation, thus providing a theoretical basis for using intracellular miR-211 as a possible anti-AS target [55]. Combining these results with our study, we propose the conclusion that curdione may facilitate the growth of ox-LDL-caused HUVECs via regulating DNMT1-mediated ERBB4 promoter methylation.

Nevertheless, curdione cannot completely reverse ox-LDL-induced adverse effects; the reasons may be due to the complexity of the ox-LDL-mediated pathophysiological pathways. ox-LDL triggers a cascade of events involving multiple cell types and signaling molecules [56,57], while curdione acts on specific targets (such as certain genes related to inflammation and lipid metabolism in endothelial cells) within this complex network [58]. Another possible reason is related to the pharmacokinetics of curdione. The drug may not achieve optimal concentrations at the site of action for a sufficient duration to completely reverse ox-LDL-induced adverse effects. In addition, cells exposed to ox-LDL for an extended period may develop adaptive or resistant mechanisms that curdione cannot fully overcome. When cells are continuously challenged by ox-LDL, they may upregulate certain survival pathways as a compensatory response. These adaptive changes can make the cells less responsive to the normalizing effects of curdione.

Additionally, epigenetic modifications may occur in cells due to long-term ox-LDL exposure. These epigenetic changes can alter gene expression patterns [59], and curdione may not be able to reverse these epigenetic marks effectively, leading to incomplete restoration of normal cellular function. Further research is needed to understand these cellular adaptation mechanisms and develop strategies to enhance curdione’s effectiveness in such resistant cell populations. The potential advantages of curdione over current AS therapies can be discussed from the perspectives of echanistic multi-target effects, reduced side-effect profiles, and the potential for combinatorial synergy. Statins primarily target LDL-C reduction via HMG-CoA reductase inhibition [60], while curdione may exert broader effects by modulating the inflammatory cytokines IL-6, IL-1β, and IL-8, the apoptosis-associated proteins Bax, Bcl-2, and caspase-3, and the EndMT-related proteins E-cadherin, F-actin, and VEGF, thereby ameliorating AS progression. Unlike monoclonal antibodies (e.g., anti-IL-1β) or colchicine (specific to NLRP3) [61,62], curdione simultaneously inhibits multiple pro-inflammatory pathways (e.g., TNF-α, IL-6, and inflammasome activation), potentially offering broader efficacy across AS stages. In addition, while DNA methylation inhibitors (e.g., 5-AZA) globally alter epigenetic marks [63], curdione may selectively modulate methylation-related enzymes (e.g., DNMTs or HDACs) in AS-relevant genes. We acknowledge that direct comparative studies between curdione and standard AS drugs are currently lacking. Future head-to-head preclinical trials and pharmacokinetic optimization (e.g., nano-formulations) will be critical to validate these hypotheses.

This study has some limitations. First, dose–response and time-course experiments should be considered to confirm the optimal condition of curdione in the future. Second, further investigations should be conducted to knock down ERBB4 to validate the dependency of the ERBB4 pathway in the efficacy of curdione. Third, the bioavailability, safety, dosage requirements, and pharmacological activities of curdione should also be further explored. Additionally, the specific mechanism of curdione upregulation of ERBB4 via DNMT1 regulation and how DNMT1 regulates ERBB4 promoter methylation (through transcriptional activation or histone modifications associated with DNMT1 activity) need to be further explored via a series of experiments, such as methylation-specific PCR or bisulfite sequencing and *in vivo* models.

## 5 Conclusion

Both curdione and ERBB4 overexpression may ameliorate AS development through the inhibition of apoptosis, inflammation, and the EndMT of HUVECs. In addition, a combination of curdione and ERBB4 overexpression could show stronger effects compared to individual administration. The possible mechanisms are closely associated with inflammatory cytokines, apoptosis-related proteins, and EndMT-related proteins. Additionally, curdione may protect the vascular endothelial cells and AS via the regulation of DNMT1-mediated ERBB4 promoter methylation. Our work lays the theoretical basis for the treatment of AS and other forms of CVD with curdione as a novel therapeutic drug and ERBB4 as a potential new therapeutic target.
